# The Swedish Health Promoting Healthcare network and the built environment

**DOI:** 10.1093/heapro/daab101

**Published:** 2021-07-09

**Authors:** Elke Miedema, Göran Lindahl, Marie Elf

**Affiliations:** Division of Architectural Theory and Method, Department of Architecture and Civil Engineering, Chalmers University of Technology, SE-412 96 Gothenburg, Sweden; Division of Building Design, Department of Architecture and Civil Engineering, Chalmers University of Technology, SE-412 96 Gothenburg, Sweden; School of Health and Welfare, Department of Nursing and Midwifery, Dalarna University, 791 88 Falun, Sweden

**Keywords:** built environment, health promoting environments, healthcare facilities, health-promoting hospitals, salutogenesis

## Abstract

The Health Promoting Hospitals (HPH) networks, founded by the World Health Organisation, support the introduction of health promotion in healthcare. This development involves the creation of a health promoting built environment. However, few studies have explored the HPH in relation to the built environments, and it is unclear how HPH-networks incorporate the built environment in their work. The study therefore examined the Swedish HPH-Network in relation to the built environment. The mixed-method study included data from (i) key online material from the Swedish network, (ii) a survey with open-ended questions of representatives of the networks’ workgroups and (iii) semi-structured interviews with the built environment workgroup. The study showed that the built environment is unevenly and incoherently incorporated in the network. Moreover, there is more attention for healing and healthy rather than health-promotive strategies, indicating a knowledge gap. Descriptions of the health promoting built environment are diverse, and address design features, design strategies or indicate places for health promotion interventions. The descriptions of the built environment are combined with various HPH goals and population groups. To utilize the built environment as a resource for HPHs, the networks should consider incorporating the built environment in documents and action plans at all organizational levels.

## INTRODUCTION

Traditionally, a healthcare focused on diagnosis, treatments and care of disease (pathogenic approach) (Antonovsky, 1996; Hancock, 2012). However, the complexity of health issues, including ageing societies, rise of chronic and non-communicable diseases, climate change and increasing health inequities, requires pro-active approaches that focus on people (rather than an illness) and keeping people healthy regardless of their abilities and age (salutogenic approach) (Antonovsky, 1996; Hancock, 1999; Wilson et al., 2011). The World Health Organization (WHO) is therefore encouraging healthcare organizations to incorporate health promotion in their structures, culture, decisions and processes (WHO Europe, 2007). Health promotion is ‘a process devoted to empowering (vulnerable) individuals and communities to take control over the factors that positively influence their health and quality of life including their social, natural and built environment’ (Miedema, 2020, p. 62). The International Network of Health Promoting Hospitals and Health Services (HPH-Network) was initiated by the WHO in 1990 with the goal to support hospitals in the process of becoming health promoting organizations (Pelikan et al., 2001; Whitehead, 2004; WHO Europe, 2006, 2007). Health promotion work, and specifically health promotion in healthcare, should include the creation of a health promoting physical environment (WHO Europe, 2007; Golembiewski, 2010; Hancock, 2012; Dietscher et al., 2017; Miedema et al., 2019b) which is the combination and interaction between the natural (air, soil, plants and water) and built environment (buildings and spaces created or modified by humans) (Schulz and Northridge, 2004). Despite the recognized importance of the physical environment for HPH, no systematic investigation has been performed on how the built environment is integrated into the HPH-networks.

As Sweden has been at the forefront of health promotion work, including the development of new healthcare environments, the analysis of the Swedish HPH-network can provide valuable information on ongoing health promotion strategies. This study therefore questions how the built environment has been incorporated into the Swedish HPH-network as described in the documentation and by HPH-network representatives. The findings can contribute to understanding of the role of HPH-network in the development of HPH built environments and thereby inform future healthcare building designs and the HPH strategies.

## BACKGROUND

HPH are hospitals and healthcare organizations that re-orient from a disease-centred care to people-centred care, that pay attention to health promotion for both patients, as well as staff, visitors, the community and the planet (Hancock, 1999, 2012; Miedema, 2020). HPH work can include attention to positive health, healthy behaviours, health equity and empowerment (Johansson et al., 2009; Miedema et al., 2019a). HPH should include all types and sizes of healthcare environments in very different health systems (Pelikan et al., 1998). These HPH are supported by international and national HPH-Networks and HPH-standards.

The Swedish HPH-network is a part of the international network and involves various healthcare representatives such as healthcare management, physicians, physiotherapists, nurses and other healthcare professionals. These representatives are involved in diverse roles, such as the steering committee or in themed workgroups. The HPH-network is presented on their website (www.hfs.se), which documents their main strategies, supportive material, members organizations and the work of the workgroups. The 13 workgroups focus on diverse topics with both preventative foci (alcohol and tobacco prevention) as well as health promotion foci (physical activity, a health promotion workplace, a health promotion approach, health promotion primary care, eating habits, patient-reported outcome measures, mental health, targeted health talks and evaluation of HPH strategy goals). One of the workgroups focuses on health promoting built environment (HPBE) (Noorlind Brage, 2017). Most Swedish healthcare regions are members of the national HPH-network (HFS-nätverket, 2014) and pay an annual fee, agreed to make efforts to incorporate health promotion strategies in their organization and agreed to follow the agreed HPH standards (HFS-nätverket, 2018).

The WHO (WHO, 1991a) has recognized the importance of the physical environment for health outcomes and health promotion, especially among vulnerable populations such as people with long-term health conditions (Nathan et al., 2018). Here, ‘environment’ refers to the inter-dependency between social–cultural, natural and built environment. The built environment consists of several features; including ambient features (e.g. light, acoustics, air quality and temperature), architectural features (e.g. plan layout, structure, room size and shape), interior (e.g. equipment, finishing, and furniture), maintenance features (housekeeping services, cleanliness and wear) and social features (privacy, control, access and familiarity) (Harris et al., 2002). The HPH standards developed by the WHO (WHO, 1991, 1997) and HPH-networks state that; ‘[t]he physical environment of hospital buildings should support, maintain and improve the healing process’; and ‘develops itself into a health promoting physical environment’ (WHO, 2004; WHO Europe, 2007; HPH-network, 2014). This means that HPH should pay attention to the built environments (Pelikan et al., 2001; Hancock, 2012; Dietscher et al., 2017) and participate in discussions about health-related outcomes of care building design (Hancock, 1999).

The healthcare built environment can influence clinical outcomes, patient and staff experiences, and the economic performance of the facility (Ulrich et al., 2010; Sadler et al., 2011; Huisman et al., 2012). Such research generates knowledge to inform the design of healthcare buildings (Ulrich et al., 2010; Hamilton, 2016). However, healthcare building design research has focused mainly on acute environments such as surgery and intensive care (Joseph et al., 2018) and with significant attention paid to residential aged care (Chaudhury et al., 2018) and mental health facilities (Connellan et al., 2013). Fewer studies focused on design perspectives for health promotion. Diverse perspectives can be found such as design for well-being, health behaviour, health equity or empowerment (Miedema et al., 2019b; Miedema, 2020). Design for well-being focuses on individuals' positive and holistic health outcomes (Miedema, 2020) and can include concepts such as salutogenic design. Golembiewski (2017) wrote about Salutogenic design of a psychiatric facilities which pays attention to the building design as source to cope with stress and support manageability, comprehension and meaning. Buildings can also limit or stimulate certain health-related behaviours, such as healthy eating, physical activity, social interaction or sleep. For example, by designing and integrating educational kitchens in a healthcare building (Miedema et al., 2019b; Miedema, 2020) or encouraging people to be more active by designing attractive stairs (Feenstra, 2021; Foster and Hillsdon, 2004). Attention to health equity and the needs and abilities of vulnerable populations, the design can be culturally sensitive, universal and inclusive. For example, improving wayfinding for people with visual disabilities (Rousek and Hallbeck, 2011) or support the specific need for people with dementia or children (Chaudhury et al., 2018). Lastly, the need for empowerment has been related to both to the ability to be involved in the design process of new environments (Eriksson et al., 2012; Elf et al., 2015), access to health information (Miedema et al., 2019a), as well as being able to control your surroundings (Golembiewski, 2010; Ulrich et al., 2010), such as the temperature or lighting.

However, a review on how health promotion design was implemented in outpatient healthcare, showed an overall lack of research, and that the research tends to be fragmented; thus focused on only a few aspect of health promotion and not combining for instance design for health equity and healthy behaviours (Miedema et al., 2019b). Moreover, there seems no consensus as to what design for health promotion means and thus what it should focus on (Miedema et al., 2019b). Previous research has also shown that professionals who work in Swedish HPH do not always agree on the importance of the built environment nor do they incorporate health promotion into building design interventions and they are not familiar with the research in the area (Miedema et al., 2019a). In addition, the representatives in the Swedish HPH-network are not aware of the HPH standards, strategies or the role of the built environment for the development of health promotion (Miedema et al., 2019a).

Despite a large and growing body of evidence linking human health to the built environment (Ulrich et al., 2010; Sadler et al., 2011; Huisman et al., 2012), and indications that the incorporation of the built environment in healthcare strategies contribute to improved healthcare quality (Anåker et al., 2017) it remains challenging to include the built environment in healthcare strategies and policy work (Lowe et al., 2014). The aim of this study was therefore to examine the Swedish HPH-Network in relation to the built environment, in their documents or as mentioned by their representatives. The research questions addressed were (i) which terminologies are used to address the built environment?, (ii) What design features are addressed?, (iii) What design strategies are addressed (e.g. components of the design process)?, (iv) What health promotion perspectives and (v) target populations are addressed?

## MATERIALS

This study was a mixed-method study that includes data from (i) key online documentation from the Swedish HPH-network, (ii) a survey with representatives of the network workgroups and (iii) interviews with members of the workgroup which focus on the built environment.

### Setting of the study

The study was performed in 2019 and focused on the Swedish HPH-network, namely represented by their public website, the steering committee and 13 workgroups.

### Participants in the survey and interviews

There were 11 participants in the survey and 3 participants in the interviews. The survey participants were the main representatives of the workgroups and were therefore expected to know the most about workgroup activities. These representatives were identified through HPH-network website. All 13 workgroups were contacted by phone before participation, and 11 responded to the survey.

For the interviews, 3 out of 10 members of the HPBE workgroup were intentionally chosen to provide additional insight into how building design was included in their work. The chosen participants were the chair of the workgroup, who should be most informed about ongoing development; one member who performed research on the design of healthcare environments; and one member who was recommended by the HPBE chair due to involvement in the HPBE workgroup and in health promotion interventions. Their contact information was found on the HPH website, and they were contacted by email by the first author.

### Data collection

All data, including the HPH-network online documents, survey data, and semi-structured interview data, were collected between December 2017 and September 2018. First, the first author identified documents from the Swedish HPH-network’s website, including 279 webpages and 198 linked documents. Those documents were retrieved from their public website and scanned to determine whether they were related to network strategies or visions and developed or shared by the HPBE workgroup. The documents were also required to address the built environment through terms such as ‘setting’, ‘physical environment’, ‘architecture’, ‘interior’ or ‘building design’. The materials included webpages with texts and images; links to external webpages and entire websites; and links to documentation, such as newsletters, presentations, reports, standard and planning and strategic material. External links were only included if they linked to specific pages not full websites.

The online survey was developed for this study to assess how the workgroups viewed the built environment in relation to their health promotion work. The survey included the following open-ended questions: what are the main strategies of the workgroup, what hospital interventions have been developed in the workgroup, in what way is the work related to the theme of the group and associated with the built environment and in what way might the built environment support or hinder health promotion? The participants could choose when, where and in what language to participate due to the survey format.

The interviews with the HPBE workgroup took 1 h and were conducted using a semi-structured interview guide developed by the research team. The questions focused on the role of the built environment in relationship to HPHs; overall goals of the workgroup, their description of a HPBE, their involvement in building projects, and their collaboration with other workgroups. The interviews were conducted by the first author (experienced with interview techniques) face-to-face in a common, familiar setting or over the phone. The respondents could answer in Swedish or English. All the interviews were audio-recorded and transcribed.

### Data analysis

The document identification, survey and interviews produced three sets of data which were analysed based upon a deductive content analysis (Elo and Kyngäs, 2008). Before we combined the data, we prepared each data set separately. The data from the documents were the text sections that addressed the built environment; those were extracted and cut into meaning units (i.e. text fragments that contain aspects related to each other in content and context (Graneheim and Lundman, 2004). The interview transcriptions were also extracted and cut into meaning units. The survey data were extracted per question and compiled into one list. All the data were then imported into one Excel template that mapped the source of the data and the original meaning units. The original meaning units were then translated into English and condensed while keeping the original meaning. The data were re-read to familiarize with the information and the first author then created six categories according to research questions (based on the work of Miedema et al., 2019b). The first author then created 28 sub-categories from the coded text which were compared, contrasted and interpreted in the research group.

### Ethics

The participants were informed verbally and in writing of the aim of the study, that their answers would be recorded, and that the data collected would only be used for this study. They were also informed that the personal information would only be known by the research team and would be protected according to the General Data Protection Regulation. Informed consent was obtained when participants agreed to complete the survey.

## RESULTS

The results showed that the built environment was noted in all data sources but to various degrees. For instance, there was little mention of the built environment in the overall strategic material from the website, while the documentation from the workgroup focused on the built environment included extensive material addressing the built environment. This documentation included diverse types of materials, including scientific papers, Master’s and Bachelor’s thesis work and links to entire external websites and pages. The data show different ways to address the built environment also relating to several interpretations of HPHs as mentioned in combination with the built environment (see Table 1).

**Table 1:** Overview of the main categories, subcategories and case examples of the built environment

Category	Subcategory	Case example
Terms being used to describe the built environment	Built environment	The built environment can support health promotion by; colours, light, entrance design and increasing the privacy by improving the acoustic situation.
	Building design	To create good architecture, you need to combine research-based knowledge with the field of care, local issues, location, organization, technology, care process, treatments, patient perspective, staff interests.
	Environment	Patient’s health can be improved by different aesthetic and environmental elements in the caring surroundings.
Specific health-promotive environments	The hospital site	Not long ago, we made a parking lot at the hospital site; from the beginning, it was supposed to be a green area.
	The hospital building	I think the first time you go [to the hospital], it should make you feel it will be good and easy to find the way.
	Patient rooms	Single-patient room helps reduce airborne and contact-related infections.
	Staff support spaces	Decentralization of workstations and placement of clean/dirty storage in direct connection with patient rooms to reduce walking and retrieval time, thereby increasing time for direct care activities.
	Healthcare spaces	Introducing positive, inspiring, and restorative themes of beautiful nature into the ward environment is related to reduced experience of stress by patients and staff.
	Supportive spaces	An inviting waiting room environment with (health) information, self-care and control.
	Circulation spaces	A design that enables good visibility of patients from corridors and workstations helps reduce falls.
	Furniture	The main basics (..) Are to have clean, nice-looking furniture without old coffee cups, damaged old newspapers, or (..) Things like that around.
Design strategies for HPH built environments	Working together	Many small interventions that together make a bigger whole.
	Incorporating research	Evidence-based design [works] on two levels: as a scientific fact base for informed design decisions and as a process to clarify decision making in the design process. Design decisions are documented, which can also support future performance measures.
	User-centred perspectives	Include the patient as well as the professional perspective.
	Priorities in design process	Health promotion perspectives may come after the initial planning stage of hospital building.
Design features for HPH built environment	Ambient aspects	The staff storage in the intensive care unit has silent locks to improve sleep at night.
	Architectural aspects	Decentralization of workstations.
	Interior aspects	Introducing positive, inspiring, and restorative themes of beautiful nature into the ward environment.
	Social aspects	To understand the healthcare context, including the audience of the artwork, the artist should spend much time with the architect and staff working there.
	Maintenance aspects	(…) We talked about more sustainability in the hospital area; we talked about ponds where [rain] water can be collected (..) [which can be used to] water the plants.
health promoting perspectives	Quality of healthcare	Attractive, appealing, or comfortable rooms increase patients’ perceived quality of care and satisfaction.
	Health protection	Single-family rooms designed to promote family presence can help reduce the number of falls.
	Prevention	A work environment with a stressful atmosphere affects both patients’ healing as well as staff working conditions negatively.
	Empowerment	[The waiting room] should be a stimulating environment but also one where you can go through literature or the web so you can be active when you are waiting there and can get more knowledge about your disease or about problems.
Target populations described	Patients	Patients’ health can be improved by different aesthetic and environmental elements in the caring surroundings.
	Staff	Creating good working environments, including conditions for person-centred meetings, even between employees.
	Other building users	Patients, staff and visitors declared that beautiful nature is valuable and healing and can give calmness and hope.
	The community	Raise awareness of the (physical) environmental impact of the hospital on the health of patients, staff and the community; the healing process should be supported, maintained and improved the healing process.

### The built environment as conceptualized in HPH-network materials

The built environment was addressed in various terms; however, none of these concepts or words, were defined or explained in the material. The terms were categorized into four different groups: the built environment, building design process, the environment (e.g. socio-physical environment) and places for health promotion.

Built environment was used only by the respondents in the interviews and survey, most likely because the word was used in the questions. However, the built environment as a concept was also addressed in terms of ‘structures’ and ‘buildings’. Building design process was used to categorize anything related to the process of designing a building, such as ‘designing a new department’, ‘architectural design’ or ‘architecture’. For instance, a respondent mentioned, ‘To create good architecture, you need to combine multiple types of knowledge’. The environment was addressed in most of the material, and this included comparable words, such as ‘setting’, ‘circumstances’, ‘surroundings’, ‘area’, or ‘conditions’. One HPBE document noted that patient health can be improved using different aesthetic and environmental elements in the care surroundings. The selected material also included information on where health promotion should take place (i.e. specific health promotion settings). These descriptions could be divided into multiple building scales, from as large as a building site and building to smaller scales, such as department units, rooms and even doors or windows. The place indications found could also be grouped according to their function, including circulation areas, care areas, supportive areas and public areas. Examples of circulation areas described are corridors, hallways, stairs, entrance and walking and cycling environments. Patient rooms, stairs, waiting areas, wards, departments, workplaces and gardens or outdoor environments were place indications that re-occurred throughout the data. For example, patient rooms were suggested in the HPBE documents and were also mentioned by an HPBE representative.

### Design strategies

The data included several descriptions of design strategies, i.e., approaches and attention points for creating an HPH built environment. These strategies were related to working together, incorporating research, user-centred perspectives and priorities in the design process. Working together was addressed by one of the HPBE representatives, who mentioned ‘[T]hat many small environmental interventions can, together, create a bigger picture’. The need to incorporate research was the most addressed strategy, which included doing one’s own research, collecting existing research and implementing research in design strategies. For example, one of the HPBE documents stated that ‘good architecture combines research-based knowledge with the field of care, local issues, the location, the organization, technology, the care process, treatments, patient perspectives, staff interests and knowledge of different design features related to clinical or experimental outcomes’. Priorities in the design process included a focus on implementation, identification of problems and prioritization of different aspects in different stages of the process. For example, an HPBE representative stated that ‘it is very easy to talk about new topics, but it is very difficult to implement new ideas and new ways of working in healthcare’. Most of these design strategies were found in the HPBE documentation and mentioned in the interviews.

### Design features

The study identified a range of design features that could be grouped according to Harris et al. (Harris et al., 2002) as ambient, architectural, interior, social and maintenance features. Ambient features in the data included acoustics, daylight and climate. Architectural features mentioned were layout, stairs and elevators, ceiling lifts and decentralized workstations. Art, furniture, computers, plants and signage were interior design features described in the network. The social features that were described were related to territoriality, privacy and contextuality. For instance, one HPBE document suggested that art in hospitals should be adjusted to healthcare contexts. There was little attention to maintenance features. One interviewee addressed the need for a clean and tidy environment, and another described the need for artwork materials that are easy to maintain. Nature was also addressed throughout the data sources through terms such as ‘parks’, ‘gardens’, ‘trees’, ‘plants’ and ‘flowers’. For example, an HPBE document noted that ‘the staff wished for more varied nature photographs and more plants’. There were no design features found in the strategic documents, and such features were observed only to a small degree in the survey data. Most design features were described in the interviews and HPBE documentation.

### Conceptualization of HPHs in combination with the built environment

The built environment was described in relation to several and diverse health promotion perspectives concerning the quality of care, health protection, prevention or health promotion. Improved quality of healthcare included outcomes related to the experience of care, treatment quality and organizational results. The experience of care was mentioned in relation to the atmosphere and attractiveness of the care environment, experienced privacy and integrity, and satisfaction with care. For instance, an HPBE document indicated that ‘an attractive waiting room is more important than a short waiting time when considering patients’ experience of good quality of care’. Design features related to improved care and treatment, mostly those related to medical treatment and cure times, were found in all sources. One respondent expressed that ‘[the] built environment can support better treatment results (…)’.

Health protection was described in terms of safety, hygiene, and environmental pollution. ‘Safety’ included patient and staff injury and treatment errors, while ‘hygiene’ referred to contamination and hand washing. One document indicated that single-family rooms designed to promote family presence could help reduce the number of falls (Ulrich, 2012). ‘Pollution’ was used not as ‘environmental pollution’ but as a term in the description of goals for the built environment. The HPBE material and the interviews addressed green hospitals and sustainability. One respondent referred to green and black roofs and solar panels.

Prevention was mentioned in terms of working conditions and the ability for restoration. Working conditions were discussed in terms of creating a pleasant workplace, reducing work pressure and increasing staff recruitment. Several sources suggested that a healthcare organization should be a role model of a healthy workplace. Restoration was specifically mentioned regarding stress reduction, calmness and improved sleep. Several sources addressed the relationship between art, nature and stress reduction. For example, one of the HPBE documents states that nature-based art reduces stress and is preferred by most patients.

Health promotion was found in all types of data sources and was noted with terms associated with behaviour, autonomy, salutogenesis or meaning. In this context, behaviour could include both proactive, healthy behaviour and unhealthy behaviour. For instance, in several interviews and survey responses, it was mentioned that people should be able to use their time in a meaningful way when waiting, such as by looking up health-related information. Healthy behaviour included physical activity, health education, meaningful conversations, nutrition and play. In particular, being physically active re-occurred throughout the sources, mostly in relation to taking the stairs (if one can). The identified behaviour intentions focused mostly on patients and staff but also on those with administrative functions and people in general. A salutogenic orientation, or rather, an approach that emphasizes the need to ‘develop towards health and does not only focus on people who are sick’, was mentioned in only one interview.

The data also related the built environment to health promotion for diverse populations, including patients, staff, other building users and the community. For instance, the patients referred to included patients with long-term conditions and patients with chronic diseases or frail health. Staff, such as employees, personnel, practitioners, nurses, healthcare professionals, administration, management or cleaning staff, were also mentioned. Patients and staff as groups were mentioned in all data sources. Other building users included populations such as visitors, relatives and older adults. Relatives were included in the HPBE documentation and the interviews, but the strategic material and survey responses did not mention relatives at all. The community was primarily mentioned in the strategic material; the HPH standards stated that HPHs ‘need to raise awareness of the impact of the physical environmental of the hospital on the health of patients, staff and the community’.

## DISCUSSION

This study showed that the Swedish HPH-network to some extent described the built environment as an aspect of health promotion work. However, the study also revealed that the built environment was not well integrated into the network's overall strategic work. The built environment was mainly mentioned in HPBE working group documents and interviews.

The built environment was addressed in different ways and to varying degrees in the documents studied. The overall strategic material hardly incorporated any discussion about the built environment. The result may mean that the built environment was not generally accepted as important for an HPH, while research has shown that the built environment is crucial for creating HPH (Pelikan et al., 2001; Hancock, 2012; Dietscher et al., 2017). In addition, research has shown that the quality of healthcare buildings can be improved when strategic plans within the hospital organization and design decisions are related and aligned with each other (Blyth and Worthington, 2010; Ryd, 2004; Elf et al., 2012, 2018). The main objective of HPH-networks is to support healthcare organizations to develop HPH settings, which includes the built environment. Thus, the networks should guide and support decision-making about the design of healthcare buildings, which will be a challenge for the Swedish HPH-network to achieve.

The study showed that there was a weak understanding of the difference between the concepts of setting, physical environment and built environment as they were used interchangeably. This can also be recognized in the European HPH standards (WHO, 2004)( which uses the term ‘the physical environment’ without a clarification of its meaning. With a positive mindset, we can assume that they mean or at least include the built environment (e.g. the building, furniture and finishing). With a pessimistic mindset, we can argue that they refer to the setting (e.g. the hospital as a physical boundary) while paying little to no attention to the built environments. The problem is, when the built environment of the HPH is not explicitly considered or described, the healthcare building design may hinder the development of an HPH (Miedema et al., 2017, 2019b). For instance, an organization may promote physical activity through providing discounted bicycles for daily commute, while not providing the spaces conditions needed to stimulate actual bike commute, such as changing room or secure bicycle parking. Either way, misunderstandings between the spatial concepts could likely be avoided by providing definitions for them (Johansson et al., 2009).

The results further showed that the Swedish HPH-network seemed to focus primarily on developing pathogenic approaches for patients and staff, while there was less attention for salutogenic approaches or efforts that focused on the community or the natural environment. Hancock (Hancock, 1999) argued that an HPH organization should complement pathogenic perspectives with salutogenic perspectives and direct efforts for all building users, as well as the local community and natural environment. A recent review indicated that diverse interpretations of health promotion create different, possibly conflicting, demands for the built environment (Miedema et al., 2019b). Diverse interpretations of an HPH organization, as found within the network, might lead to similar issues. The network would therefore benefit from reconsidering their descriptions of health promotion and HPHs within the network at large, as well as in the different workgroups. For instance, by relating to others who already describe health promotion definitions, criteria and strategies for health promotion (Green et al., 1999) and HPH (Hancock, 1999, 2012).

The results showed a lack of interdisciplinary collaboration within and between the various working groups. For example, the people in the HPBE working group did not co-operate with the other working groups, such as those who focused on physical activity or smoke prevention. A recent study has shown that professionals working with health promotion felt that they lacked the necessary knowledge to relate their work to the built environment (Miedema et al., 2019a). Although HPH representatives are involved in healthcare building projects, they do not always link their involvement in design projects to their responsibility for health promotion; rather, they saw themselves as merely healthcare staff (Miedema et al., 2019a). The network should therefore improve the cooperation between those who pay attention to the built environment as part of the HPH work (e.g. healthcare management and employees) work and those who are involved in designing healthcare buildings (e.g. building designers and facility management).

In addition, the results showed that the HPBE representatives who were asked to be involved in design projects were involved late in the process and only within the organizations where they worked. Thus, this study underlines the importance of inter-disciplinary collaboration in health promotion building design, which is also highlighted in health promotion criteria (Green et al., 1999), the development of health promotion settings (Pelikan et al., 2001; Whitehead, 2004), and in the process of designing healthcare buildings (Elf *et al.*, 2015). Earlier involvement and connections throughout the country may increase the contribution of the built environment to HPH. Nevertheless, involvement alone does not solve the difficulties, earlier studies have shown the importance of profound knowledge of the multiple dimensions of health promotion, the diverse vocabularies and how both affect a building project (Golembiewski, 2017; Miedema, 2020; Miedema *et al.*, 2019a,b). Collaboration should therefore include clarifying ambitions and interpretations of HPH and it’s built environment, and part of both continues discussions on planning and designing of healthcare buildings, as well as HPH strategies. Improved collaboration is likely to contribute to more effective and efficient interaction between the built environment, health promotion and the objectives of HPHs.

The studies’ explorative approach, using first- and second-hand data, requires considering the findings in the study’s context (Jaeger and Halliday, 1998). The descriptions of the built environment in the Swedish HPH-network or organizations therefore cannot result in general statements about other HPH-networks and organizations. Using interviews and survey assumes that the situation can be understood from the participants’ words (Silverman, 2000; Uwe Flick, 2014). The documentation was included to trace the network’s organizational processes (Bowen, 2009; Coffey, 2014), practices and actions (Prior, 2003). The findings may inform other HPH-networks and organizations, and results provide in-depth knowledge of the chosen contexts, with suggestions for their practices and future research.

## CONCLUSION

It is essential for HPH-networks, as drivers of HPH development, to pay attention to the built environment and incorporate this in their documentation. However, this study indicates that the built environment is not well incorporated in the practices of the Swedish HPH-network and confirms a general lack of knowledge concerning HPBE. The HPH-network should instead emphasize the importance of the built environment across all organizational levels, including the strategy documentation and diverse workgroups. Second, the network should explain what is meant with terms such as ‘setting’, ‘physical environment’ and ‘built environment’ and use the terms consistently throughout its materials. Third, the network needs to clarify their interpretation of HPH; which should include a salutogenic orientation, emphasis on community health outcomes and environmental impact.

The HPBE workgroup could drive the incorporation of the built environment throughout the network and the other workgroups by providing coherent content that is easy to navigate. Their material should probably be limited to material that relates to the built environment and HPHs, rather than general healthcare. Future studies should explore the incorporation of the built environment in other HPH-networks, such as the European network.
